# Resistant Hypertension On Treatment (ResHypOT): sequential nephron blockade compared to dual blockade of the renin-angiotensin-aldosterone system plus bisoprolol in the treatment of resistant arterial hypertension – study protocol for a randomized controlled trial

**DOI:** 10.1186/s13063-017-2343-3

**Published:** 2018-02-12

**Authors:** Elizabeth do Espirito Santo Cestário, Letícia Aparecida Barufi Fernandes, Luiz Tadeu Giollo-Júnior, Jéssica Rodrigues Roma Uyemura, Camila Suemi Sato Matarucco, Manoel Idelfonso Paz Landim, Luciana Neves Cosenso-Martin, Lúcia Helena Bonalume Tácito, Heitor Moreno Jr., José Fernando Vilela-Martin, Juan Carlos Yugar-Toledo

**Affiliations:** 1 Hypertension Clinic, Department of Internal Medicine, State Medical School of São José do Rio Preto (FAMERP), Rua Tocantins 2971 Votuporanga, São Paulo, CEP 15505-188 Brazil; 2 Endocrinology Division of the Internal Medicine Department, State Medical School of São José Rio Preto (FAMERP), São Paulo, Brazil; 3 Cardiovascular Pharmacology Laboratory, Faculty of Medical Sciences, State University of Campinas (UNICAMP), Campinas, Brazil

**Keywords:** Resistant hypertension, Natriuretic agents, Dual blockade of the renin-angiotensin system, Bisoprolol

## Abstract

**Background:**

Resistant hypertension is characterized when the blood pressure (BP) remains above the recommended goal after taking three antihypertensive drugs with synergistic actions at their maximum recommended tolerated doses, preferably including a diuretic. Identifying the contribution of intravascular volume and serum renin in maintaining BP levels could help tailor more effective hypertension treatment, whether acting on the control of intravascular volume or sodium balance, or acting on the effects of the renin-angiotensin-aldosterone system (RAAS) on the kidney.

**Methods/design:**

This is a randomized, open-label, clinical trial is designed to compare sequential nephron blockade and its contribution to the intravascular volume component with dual blockade of the RAAS plus bisoprolol and the importance of serum renin in maintaining BP levels. The trial has two arms: sequential nephron blockade versus dual blockade of the RAAS (with an angiotensin converting enzyme (ACE) inhibitor plus a beta-blocker) both added-on to a thiazide diuretic, a calcium-channel blocker and an angiotensin receptor-1 blocker (ARB). Sequential nephron blockade consists in a progressive increase in sodium depletion using a thiazide diuretic, an aldosterone-receptor blocker, furosemide and, finally, amiloride.

On the other hand, the dual blockade of the RAAS consists of the progressive addition of an ACE inhibitor until the maximum dose and then the administration of a beta-blocker until the maximum dose. The primary outcomes will be reductions in the systolic BP, diastolic BP, mean BP and pulse pressure (PP) after 20 weeks of treatment. The secondary outcomes will evaluate treatment safety and tolerability, biochemical changes, evaluation of renal function and recognition of hypotension (ambulatory BP monitoring (ABPM)). The sample size was calculated assuming an alpha error of 5% to reject the null hypothesis with a statistical power of 80% giving a total of 40 individuals per group.

**Discussion:**

In recent years, the cost of resistant hypertension (RH) treatment has increased. Thus, identifying the contribution of intravascular volume and serum renin in maintaining BP levels could help tailor more effective hypertension treatment, whether by acting on the control of intravascular volume or sodium balance, or by acting on the effects of the RAAS on the kidney.

**Trial registration:**

Sequential Nephron Blockade vs. Dual Blockade Renin-angiotensin System + Bisoprolol in Resistant Arterial Hypertension (ResHypOT). ClinicalTrials.gov, ID: NCT02832973. Registered on 14 July 2016. First received: 12 June 2016. Last updated: 18 July 2016.

**Electronic supplementary material:**

The online version of this article (doi:10.1186/s13063-017-2343-3) contains supplementary material, which is available to authorized users.

## Background

Systemic hypertension is a multifactorial and complex disease that is characterized by constantly high blood pressure (BP). It is associated with functional and structural changes in target organs (heart, brain, kidneys and blood vessels) [1, 2] and metabolic abnormalities, which also increase the risk of fatal and non-fatal cardiovascular events [3].

Hypertension is a major modifiable risk factor for cardiovascular disease and one of the most important public health problems. As the BP rises above 115/75 mmHg, the mortality rate due to cardiovascular disease increases linearly [4]. The exact relationship between the number of drugs taken and the control of hypertension are unknown, although data from the Anti-Lipid Lowering Heart Attack Trial (ALLHAT) study, which prospectively followed 40,000 patients, showed that 49% of patients had their BP controlled with one or two drugs; the other 51% required three or more drugs to achieve recommended targets [5, 6].

There has been a rise in the prevalence of hypertension in recent years due to the epidemic of obesity, increased longevity and the higher incidence of kidney disease in the population [7]. On average, hypertension affects 30% of the adult population, that is, about 1.2 billion people worldwide [8]. In Brazil, 14 population studies between 1994 and 2009 have shown insufficient BP control in about 19.6% of subjects [9].

Resistant hypertension (RH) is characterized by the BP remaining above the recommended goal after taking three antihypertensive drugs with synergistic actions at maximum recommended tolerated doses, preferably including a diuretic, for at least 6 months, or on using four or more antihypertensive drugs even if the BP is controlled [10]. True RH should be differentiated from pseudoresistance, which occurs due to non-adherence to treatment, inadequate BP measurements, inadequate doses of medications, inappropriate therapeutic regimens, or the presence of the so-called white-coat effect [11–22]. For the investigation and monitoring of RH, the First Brazilian Position on RH recommends that causes of pseudoresistance, secondary hypertension and the possible use of drugs and substances that increase BP should be excluded, and high BP measurements should be checked, with special attention being paid to adherence to treatment [23].

The true prevalence of hypertension is unknown. In controlled randomized studies with thousands of hypertensive patients, approximately 25 to 30% of participants did not achieve the BP goal recommended by guidelines despite receiving three or more antihypertensive drugs; these studies included careful assessments of patient adherence to therapy and even ambulatory BP monitoring (ABPM), which identifies patients with pseudoresistance [24].

However, observational data from the North American National Health and Nutrition Examination Survey (NHANES) collected in 2003–2008 showed that the prevalence of RH among adults diagnosed with hypertension was 8.9% and among adults on antihypertensive treatment, it was 12.8% [18, 20, 25]. Similarly, a large population study in Spain (68,000 patients) found that the prevalence of RH was 14.8% among those treated for hypertension. Based on these recent studies, it is justifiable to say that the prevalence of RH is about 14% [24].

RH is a difficult-to-manage clinical condition because of patients’ failure to adhere to treatment, the physician’s difficulty to adjust the medication due to genetic factors that hinder the effectiveness of treatment and due to medical inertia [26]. The challenge lies in building an effective regimen in terms of blocking most of the implicated and individualized pathophysiological pathways according to patient profile, lifestyle, comorbidities and even financial limitations. In addition, the optimal combination should be well tolerated by the patient, with minimal adverse events to ensure long-term adherence to therapy [27].

Interventions with three different classes of antihypertensive agents, including a diuretic at the ideal dose, are necessary to achieve target values of BP in resistant hypertensive patients [10, 15, 20, 27, 28]. However, some resistant hypertensive patients, despite treatment with a three-drug regimen need at least four antihypertensive agents to gain adequate BP control [11, 13, 29, 30].

Regarding the recommendations on research priorities published by Professor Iain Chalmers, [31] we can state that studies of the pathophysiology of RH emphasize persistent fluid retention, increased sodium sensitivity, excessive salt intake, hyperaldosteronism and a certain degree of renal dysfunction as common underlying causes that contribute to the hypervolemic state found in these patients [15, 28, 32–37]. On the other hand, RH patients may present different pathophysiological mechanisms in terms of etiology and so consistently demonstrated sympathetic nervous hyperactivity as evidenced by the measurement of 24-h urinary metanephrines, increased resting heart rate (HR) mainly during sleep, increased HR variability during 24-h spectral analysis with Holter monitoring, increased arterial stiffness inferred by pulse wave velocity and increased peripheral arterial resistance [38, 39]. These markers of increased sympathetic activity together with other factors, such as hyperaldosteronism and increased renin angiotensin activity [40–45], are mechanisms that maintain high BP.

A systematic research has already been performed to assess the benefits and harms of adding a new drug to the current triple-drug regimen for management of RH in adults versus continuation of treatment with triple-drug therapy alone. Charan et al. reviewed the pharmacotherapy for RH in adults [27].

Finally, academic literature projects with similar designs but using other drugs have been published; for example, “Sequential nephron blockade versus sequential renin-angiotensin system blockade in resistant hypertension: a prospective, randomized, open, blinded-endpoint study” [46]; and “True antihypertensive efficacy of sequential nephron blockade in patients with resistant hypertension and confirmed medication adherence” [47].

However, a study of a Brazilian population and the use of other drugs not tested in these studies reinforces the importance of our study.

### Pathophysiology of resistant hypertension

The mechanisms involved in the pathophysiology of RH are vascular smooth muscle tone and increased blood volume, intensified sympathetic system activity and hyperactivity of the renin-angiotensin-aldosterone system (RAAS) [14, 21, 32, 38, 39, 43, 48, 49].

Increased sensitivity to sodium appears to be the main factor in the pathophysiology of this syndrome, not only as it mediates the above mechanisms, but also as it explains, in part, the variability of therapeutic response in patients with RH [34]. The RAAS is vital to the regulatory system that controls total body sodium, as are atrial natriuretic peptide factors and pressure receptors in the atria and kidney. Sodium and water retention can lead to resistance to antihypertensive drugs.

From the physiological point of view, both of normal subjects and hypertensive patients, BP is maintained by the continuous regulation of cardiac output and peripheral vascular resistance exerted at three anatomic sites: the arterioles, post-capillary venules (capacitance vessels) and the heart. A fourth anatomical site of control, the kidney, contributes to the maintenance of BP by regulating intravascular volume [50, 51]. The autonomic control of BP involves the baroreflex mediated by efferent fibers in the central nervous system acting on the heart and blood vessels; this activation regulates BP in tandem with humoral mechanisms with the activation of the RAAS [52, 53]. The BP is controlled by the same mechanisms in both normotensive and hypertensive subjects.

Regulation in hypertension differs from the regulation in healthy individuals as the baroreceptors and renal control systems of blood volume seem to set the BP at a higher level. Thus, identifying the contribution of blood volume and serum renin in maintaining BP levels could help tailor more effective hypertension treatment, whether by acting on the control of blood volume, the sodium balance, or by acting on the effects of the RAAS on the kidney [12, 28, 35, 50].

Sequential nephron blockade consists of progressive increases in sodium depletion. After the administration of a thiazide diuretic (chlorthalidone) and an aldosterone-receptor blocker, low doses of furosemide are administered and ultimately amiloride is prescribed to enhance the natriuretic effect [46].

Blockade of the RAAS is to increase the effect of the angiotensin receptor-1 blocker (ARB). Therapy then requires sequentially adding an angiotensin-converting enzyme (ACE) inhibitor to reduce the levels of angiotensin (Ang) II by blocking its receptor and then administering a beta-blocker to decrease the elevated renin secretion due to both the ACE inhibitors and ARBs [54, 55].

### Research questions

The following research questions will be explored:

- Does sequential nephron blockade and dual blockade of the RAAS plus bisoprolol constitute good therapeutic options in the reduction of peripheral BP of patients with RH?
- Which therapeutic option is able to reduce the central pressure in resistant hypertensive patients?
- Does non-inferiority testing demonstrate that sequential nephron blockade has the same therapeutic efficacy as dual blockade of the RAAS plus bisoprolol?

### Objectives

This study will compare two antihypertensive treatment regimens in RH patients at the Medical School in Sao Jose do Rio Preto. It aims to demonstrate the therapeutic efficacy of sequential nephron blockade compared to the dual blockade of the renin-angiotensin system plus bisoprolol in RH patients, and to assess the side effects and adherence to treatment over 20 weeks.

## Methods/design

### Study design

Allocation: randomized (blinding participants, personnel)

Intervention model: parallel assignment

Masking: none (open label)

Primary purpose: treatment

This is an open-label, prospective, randomized clinical trial (ClinicalTrials.gov, identifier: NCT02832973, registered on 18 July 2016). Two therapeutic regimens for RH will be compared: sequential nephron blockade and dual blockade of the RAAS plus bisoprolol. This study is being developed in the Medical School in Sao Jose do Rio Preto.

All participants are required to give written informed consent. The study participants are randomly allocated either to the sequential nephron blockade group or to the dual blockade of the RAAS group. All participants receive complete basic treatment.

**Fig. 1:**
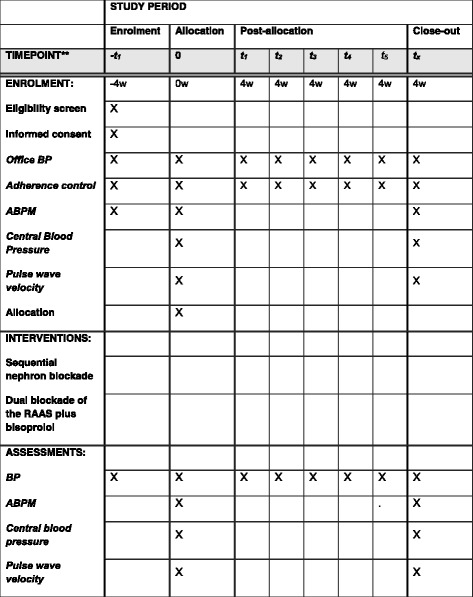
Sequential nephron blockade compared to dual blockade of the renin-angiotensin-aldosterone system plus bisoprolol in the treatment of resistant arterial hypertension: study protocol for a randomized controlled trial. Schedule of enrollment, interventions and assessments

Standard Protocol Items: Recommendations for Interventional Trials (SPIRIT) 2013 Checklist: recommended items to address in a clinical trial protocol and related documents, is available online for this manuscript (Additional file 1). The SPIRIT Figure for the trial is shown in Fig. 1.

### Participants

We are recruiting trial participants as follows:

- Patients referred to the hypertension clinic by other sectors
- Respondents to advertisements in newspapers and magazines
- Patients who have received treatment at the undergraduate student treatment clinics of the Medical School in Sao Jose do Rio Preto

### Inclusion criteria

**Fig. 2:**
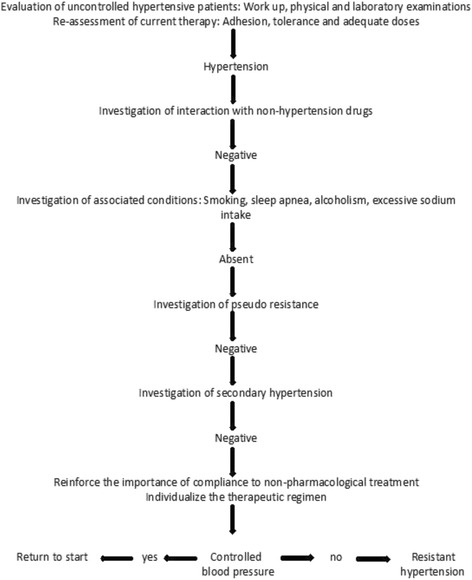
Flowchart for the diagnosis of resistant hypertension (RH) of the First Brazilian Position on RH

The inclusion criteria are as follows:

1. Men and women aged between 18 and 75 years old
2. Patients with RH identified after treatment with three antihypertensive drug classes at maximum tolerated doses for at least 6 months
3. The eligibility criteria will follow those shown in the flowchart for the diagnosis of RH of the First Brazilian Position on RH (Fig. 2)

Patients will be analyzed during five visits at 28-day intervals over 20 weeks.

### Exclusion criteria

The exclusion criteria are as follows:

1. Chronic renal failure with dialysis or creatinine clearance < 40 mL/min
2. Coronary artery disease – unstable angina; recent myocardial infarction
3. Atrial fibrillation or atrioventricular block
4. Contraindication or intolerance to the drugs that will be used
5. Refusal or failure to follow regimen
6. Secondary hypertension

### Randomization

We generated the two comparison groups using simple randomization, with an equal allocation ratio, by referring to a table of random numbers. The study coordinator will organize and number the envelopes, which will be allocated in order of patient enrollment. We develop and monitor the allocation process to preserve concealment. We use sequentially numbered, opaque, sealed envelopes. The envelopes are opened sequentially but only after the envelope has been irreversibly assigned to the participant.

Eighty patients undergoing RH treatment with losartan (100-200 mg), chlorthalidone (25 mg) and amlodipine (5 mg) will be enrolled and randomly allocated to one of two groups:

- Forty patients will receive in addition to the basal therapy, spironolactone (25 mg), spironolactone 25 mg plus furosemide (20 mg), spironolactone plus furosemide (40 mg) and spironolactone (25 mg) plus furosemide (40 mg) plus amiloride (5 mg), sequentially
- Forty patients will receive, in addition to the basal therapy, ramipril (5 mg), ramipril (10 mg), ramipril (10 mg) plus bisoprolol (5 mg) and ramipril (10 mg) plus bisoprolol (10 mg), sequentially

To achieve adequate participant enrollment to reach target sample size, we will use the written and spoken media to identify volunteers for the study.

### Interventions

**Fig. 3:**
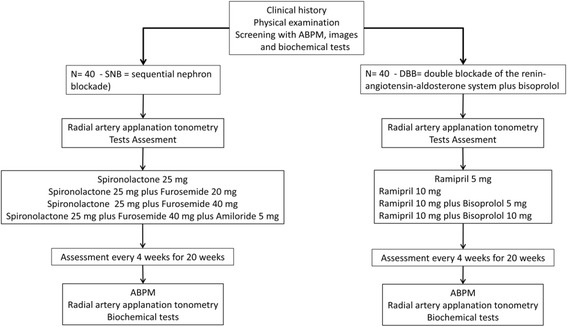
Flowchart of the study

Both groups will be analyzed in five visits at 28-day intervals over 20 weeks. Figure 3 shows a flowchart of the selection of participants and interventions.

### Randomization and follow-up

#### Protocol

**Fig. 4:**
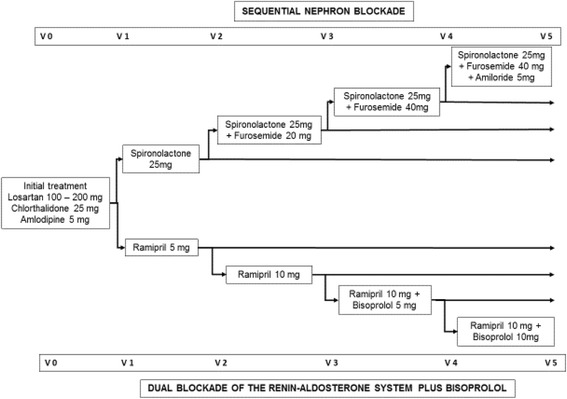
Study design

Patients will be analyzed in five sequential visits with 28 days between visits. Figure 4 shows the flowchart of the study.

- *V0*: week − 4 to week 0. All patients will remain under treatment with losartan (100–200 mg), chlorthalidone (25 mg) and amlodipine (5 mg)
- *V1*: week 0 to week 4. Individuals with BP > 135/85 mmHg by ABPM will be randomized to one of the study groups
- *V2*: week 4 to week 8. Patients randomized to one of the study groups will receive 25 mg of spironolactone (SNB group) or 5 mg of ramipril (DBB group)
- *V3*: week 8 to week 12. Individuals with BP < 135/85 mmHg by ABPM will continue using the same regimen. Subjects with BP > 135/85 mmHg by ABPM will receive, in addition to their existing regimen, furosemide (20 mg) for the SNB group and ramipril (10 mg) for the DBB group
- *V4*: week 12 to week 16. Subjects with BP < 135/85 mmHg by ABPM will continue on the same regimen. Individuals with BP > 135/85 mmHg by ABPM will receive 40 mg furosemide for patients in the SNB group and 5 mg bisoprolol for patients in the DBB group
- *V5*: week 16 to week 20. Subjects with BP < 135/85 mmHg by ABPM will continue using the same regimen. Individuals with BP > 135/85 mmHg by ABPM will receive an extra 5 mg amiloride for patients in the SNB group and 10 mg bisoprolol for patients in the DBB group
- *VEnd*: week 20 to week 24. Blood samples will be drawn from all patients. Radial artery applanation tonometry and ABPM will be performed

### Measurement of blood pressure including 24-h ambulatory blood pressure monitoring

The BP will be measured by the indirect method following the VI Brazilian Guidelines for the Treatment of Hypertension [56].

ABPM and home BP measurements (HBPM) will be carried out as additional tools to investigate hypertension. Whenever possible, the measurement of BP outside the office is recommended for a definite diagnosis because of white-coat and masked hypertension. ABPM is a method that allows the indirect and intermittent recording of BP for 24 h while patients perform their usual activities during the day. BPs equal to or greater than 130/80 (mean 24-h ABPM), 135/85 (daytime) and 120/70 mmHg (nighttime) are considered abnormal [57]. ABPM will be performed using the Mobil-O-Graph NG (I.E.M. GmbH, Cockerillstraβe, 69; 5222, Stolberg, Germany).

Monitoring requires patients to maintain their normal daily activities with the BP being measured automatically at 30-min intervals for an entire 24-h period according to the technical norms of the 5th Guidelines on Ambulatory Blood Pressure Monitoring. The systolic BP (SBP) and diastolic BP (DBP) will be obtained by ABPM with the mean values for the 24-h period, daytime and nighttime being considered for analysis. Patients with mean BP values ≥ 130/80 mmHg over 24 h, ≥ 135/85 mmHg during wakefulness and ≥ 120/70 mmHg when asleep will be considered RH. Pulse pressure (PP) will be calculated during the periods (24 h, daytime and nighttime) using the formula PP = SBP − DBP. The normal nocturnal dip will be defined as a drop of > 10% in SBP from wakefulness to the period of sleeping.

HBPM, performed by the patient or other trained person, is an indirect record of the BP that involves three measurements using validated devices in the morning and three at night for 5 days at home or at work.

### Anthropometric measurements

Weight and height, measured by anthropometric scales, will be used to calculate the Body Mass Index (BMI) using the formula BMI = weight (kg)/height squared (m^2^). BMIs of 18.5 to 24.9 kg/m^2^ are considered eutrophic values, while individuals with BMIs of 25.0 to 29.9 kg/m^2^ are overweight and ≥ 30 kg/m^2^ are obese. The abdominal circumference, measured at the midpoint between the iliac crest and the lower costal margin, is the most representative anthropometric index of intra-abdominal fat and the simplest reproducible measurement. Values equal to or below 80 cm and 94 cm are considered appropriate for women and men, respectively.

### Biochemical and imaging tests

Blood samples will be drawn from all patients at the first and last visits after fasting for 12 h to measure serum total cholesterol, high-density lipoprotein cholesterol (HDLc), low-density lipoprotein cholesterol (LDLc), very low-density lipoprotein cholesterol (VLDLc), triglycerides (TG), glucose, insulin, creatinine, sodium and potassium. The following values are considered the normal ranges: total cholesterol < 200 mg/dL, HDLc > 40 mg/dL for men and > 50 mg/dL for women, LDLc < 130 mg/dL and TG < 150 mg/dL. The LDLc fraction is calculated using the Friedewald formula (LDLc (mg/dL) = TC − HDLc − TG/5 (for TG < 400 mg/dL)). The diagnosis of diabetes is confirmed by two glycemic measurements ≥ 126 mg/dL after fasting for at least 8 h.

All patients will undergo electrocardiography, echocardiography, carotid Doppler ultrasound, ultrasound with Doppler of the renal arteries, stress testing and radial artery applanation tonometry (AT). Table 1 shows a summary of the key practical aspects of the study with all follow-up visits and requested examinations.

**Table 1:** Key practical aspects of the study with all the clinical visits and the requested exams

Visits	V0	V1	V2	V3	V4	V5	V End
Informed consent	X						
Inclusion and exclusion criteria	X						
Medical history	X						
Medical evaluation/physical examination (BP measure)	X	x	x	x	x	x	X
Randomization		x					
Creatinine	X						X
Fasting glucose	X						X
Glycated hemoglobin	X						X
Potassium	X						X
Uric acid	X						X
Total cholesterol	X						X
HDL-c	X						X
Triglycerides	X						X
Urinary sodium	X						X
Microalbuminuria	X						X
GFR estimation	X						X
Specific biochemistry tests	X						X
ABPM	X						X
ECG	X						X
Radial artery applanation tonometry		x					X
Images tests	X						

*ABPM* ambulatory BP monitoring, *BP* blood pressure, *ECG* electrocardiogram, *GFR* glomerular filtration rate, *HDL-c* high-density lipoprotein cholesterol

### Primary outcome measures

Office-measured SBP and DBP at week 20, an average of three measurements using an oscillometric device (Time frame: at week 20).

### Secondary outcome measures

Efficacy: office-measured mean blood pressure (MBP) at week 20, an average of three measurements using an oscillometric device (time frame: at week 20).

Efficacy: office-measured pulse pressure (PP) at week 20, calculated from an average of three measurements using an oscillometric device (Time frame: at week 20).

Efficacy: mean 24-h SBP and DBP at week 20 measured with an ABPM device (Time frame: at week 20).

Safety and tolerability: (Time frame: during the study).

During the study, BP will be evaluated every 4 weeks by office-measured BP measurement in order to detect hypotension) (Time frame: every 4 weeks).

### Assessment of outcomes

Blood pressure (mean of three measurements by an automatic electronic device Omron HEM-711 DLX) and hemodynamic parameters (by Omron HEM 9000 AI device) will be measured in the office during follow-up visits.

In order to improve adherence to intervention protocols, we use drug tablet return and laboratory tests to monitor patient compliance.

### Adverse events

Analysis of safety-related data will be performed with respect to frequency of serious adverse events (SAEs) stratified by causality and intensity of morbidity in both treatment groups. Patients will be interviewed at each visit about the occurrence of any adverse events, including time of onset, duration and severity; all information will be recorded on a Case Report Form. The causal relation to the study drug and the intensity of adverse events will be evaluated by the investigators. SAEs must be reported to the Institutional Review Board and study sponsor by the principal investigator within 24 h after the SAE becomes known.

Laboratory adverse events, such as metabolic changes and glomerular filtration rate, will be analyzed at the final visit of patients.

### Missing or dropout

Participants will be registered with a phone number and address for further contact in case they miss scheduled visits.

Furthermore, all participants are requested to promptly report possible adverse events by telephone. Study participants receive telephone contact numbers from the study team at the time of inclusion (visit 0).

### Withdrawal of trial participants

Participants can withdraw from the trial at any time for any reason without their medical care being affected.

Data already collected will continue to be used, and the patients will be asked if they are still willing to provide follow-up data. The reason for withdrawal will be documented whenever possible.

### Application of washout?

No washout period will be used.

### Sample size

Eighty eligible patients undergoing RH treatment with losartan (100–200 mg), chlorthalidone (25 mg) and amlodipine (5 mg) will be enrolled and randomly allocated into one of two groups.

### Sample size calculation

The site https://www.stata.com/features/power-and-sample-size/ and Stata 11 program were used to estimate the sample size. The sample size was calculated at 36 patients per group (SNB versus DBB) considering an alpha error of 5%, statistical power of 80%, standard deviation (SD) of 8 mmHg, and maximum acceptable absolute difference of 6 mmHg (diastolic BP). However, considering a potential 10–15% dropout or loss to follow-up rate, 40 patients will be enrolled in each group. The difference of 5 mmHg (diastolic) has been achieved, on average, in clinical trials that have demonstrated the advantage of a drug over placebo or other non-pharmacological treatments in the prevention of major cardiovascular outcomes.

### Statistical analysis

The *t* test or Wilcoxon test for quantitative variables and the chi-square test and Fisher’s exact test for qualitative variables will be used in the comparative analysis of the clinical characteristics of RH patients. Data will be expressed as means ± 1 SD.

The sample size was estimated at 72 individuals for an expected zero difference with a SD of 12 mmHg to demonstrate the non-inferiority of the strategy of sequential nephron blockade compared to dual blockade of the RAAS plus bisoprolol assuming an absolute difference of ≤ mmHg for systolic BP.

Non-inferiority will be evaluated for a one-sided 95% confidence interval (CI) estimated by a linear mixed model for repeated measures. *P* values < 0.05 will be considered statistically significant.

## Discussion

The aims of this study comparing two antihypertensive treatment regimens in patients with RH are:

1. To demonstrate that the pharmacological treatment with sequential nephron blockade has the same antihypertensive efficacy as dual blockade of the RAAS plus bisoprolol after 20 weeks of active treatment of patients with RH.
2. To evaluate the clinical and biological safety of sequential nephron blockade compared to dual blockade of the RAAS plus bisoprolol over 20 weeks of active treatment.
3. To assess the side effects and adherence of sequential nephron blockade compared to dual blockade of the RAAS plus bisoprolol over 20 weeks of active treatment.

### Trial status at the time of initial manuscript submission

At the time of manuscript submission, 50% of participant recruitment had been completed.

Estimated enrollment: 80 including estimated 10% loss to follow-up.

Study start date: September 2014.

Study completion date: December 2017.

Work in progress, still recruiting, not finalized. To date we have 61 patients included.

## Additional files

Additional file 1: Free and clear term-compliance for research project's participation. (JPG 75 kb)

Additional file 2: Research Ethics Committee. Approval Nº 870 093. (PDF 180 kb)

## Abbreviations

ABPM: Ambulatory blood pressure monitoring
ACE: Angiotensin-converting enzyme
ALLHAT: Anti-Lipid Lowering Heart Attack Trial
Ang II: Angiotensin II
ARB: Angiotensin receptor-1 blocker
BP: Blood pressure
DBB: Dual blockade of the renin-angiotensin-aldosterone system plus bisoprolol
HBPM: Home blood pressure measurement
HDLc: High-density lipoprotein cholesterol
LDLc: Low-density lipoprotein cholesterol
NHANES: National Health and Nutrition Examination Survey
RAAS: Renin-angiotensin-aldosterone system
RH: Resistant hypertension
SNB: Sequential nephron blockade
TG: Triglycerides
VLDLc: Very low-density lipoprotein cholesterol

## References

[1] Kannel WB (1996). Blood pressure as a cardiovascular risk factor: prevention and treatment. JAMA.

[2] Kannel WB (2000). Risk stratification in hypertension: new insights from the Framingham Study. Am J Hypertens.

[3] Karmali KN, Lloyd-Jones DM (2017). Global risk assessment to guide blood pressure management in cardiovascular disease prevention. Hypertension.

[4] Lewington S, Clarke R, Qizilbash N, Peto R, Collins R (2002). Age-specific relevance of usual blood pressure to vascular mortality: a meta-analysis of individual data for one million adults in 61 prospective studies. Lancet.

[5] Major outcomes in high-risk hypertensive patients randomized to angiotensin-converting enzyme inhibitor or calcium channel blocker vs diuretic: The Antihypertensive and Lipid-Lowering Treatment to Prevent Heart Attack Trial (ALLHAT). JAMA. 2002;288(23):2981-97. 10.1001/jama.288.23.2981 12479763

[6] Bangalore S, Davis BR, Cushman WC, Pressel SL, Muntner PM, Calhoun DA (2017). Treatment-resistant hypertension and outcomes based on randomized treatment group in ALLHAT. Am J Med.

[7] Wong ND, Lopez VA, L’Italien G, Chen R, Kline SE, Franklin SS (2007). Inadequate control of hypertension in US adults with cardiovascular disease comorbidities in 2003–2004. Arch Intern Med.

[8] Prince MJ, Ebrahim S, Acosta D, Ferri CP, Guerra M, Huang Y (2012). Hypertension prevalence, awareness, treatment and control among older people in Latin America, India and China: a 10/66 cross-sectional population-based survey. J Hypertens.

[9] Rosario TM, Scala LC, Franca GV, Pereira MR, Jardim PC (2009). Prevalence, control and treatment of arterial hypertension in Nobres – MT. Arq Bras Cardiol.

[10] Calhoun DA, Jones D, Textor S, Goff DC, Murphy TP, Toto RD (2008). Resistant hypertension: diagnosis, evaluation, and treatment. A scientific statement from the American Heart Association Professional Education Committee of the Council for High Blood Pressure Research. Hypertension.

[11] Siddiqui M, Calhoun DA (2017). Refractory versus resistant hypertension. Curr Opin Nephrol Hypertens.

[12] Cai A, Calhoun DA (2017). Resistant hypertension: an update of experimental and clinical findings. Hypertension.

[13] Siddiqui M, Dudenbostel T, Calhoun DA (2016). Resistant and refractory hypertension: antihypertensive treatment resistance vs treatment failure. Can J Cardiol.

[14] Dudenbostel T, Siddiqui M, Oparil S, Calhoun DA (2016). Refractory hypertension: a novel phenotype of antihypertensive treatment failure. Hypertension.

[15] Calhoun DA (2016). Refractory and resistant hypertension: antihypertensive treatment failure versus treatment resistance. Korean Circ J.

[16] Modolo R, de Faria AP, Sabbatini AR, Barbaro NR, Ritter AM, Moreno H (2015). Refractory and resistant hypertension: characteristics and differences observed in a specialized clinic. J Am Soc Hypertens.

[17] Boswell L, Pascual J, Oliveras A (2015). Resistant hypertension: do all definitions describe the same patients?. J Hum Hypertens.

[18] Vongpatanasin W (2014). Resistant hypertension: a review of diagnosis and management. JAMA.

[19] Modolo R, de Faria AP, Sabbatini AR, Moreno H (2014). Resistant hypertension revisited: definition and true prevalence. J Hypertens.

[20] Calhoun DA, Booth JN, Oparil S, Irvin MR, Shimbo D, Lackland DT (2014). Refractory hypertension: determination of prevalence, risk factors, and comorbidities in a large, population-based cohort. Hypertension.

[21] Sim JJ, Bhandari SK, Shi J, Liu IL, Calhoun DA, McGlynn EA (2013). Characteristics of resistant hypertension in a large, ethnically diverse hypertension population of an integrated health system. Mayo Clin Proc.

[22] Moreno H, Coca A (2012). Resistant and refractory hypertension: reflections on pathophysiology and terminology. Blood Press.

[23] Alessi A, Brandao AA, Coca A, Cordeiro AC, Nogueira AR, de Magalhaes DF (2012). First Brazilian position on resistant hypertension. Arq Bras Cardiol.

[24] de la Sierra A, Segura J, Banegas JR, Gorostidi M, de la Cruz JJ, Armario P (2011). Clinical features of 8295 patients with resistant hypertension classified on the basis of ambulatory blood pressure monitoring. Hypertension.

[25] Persell SD (2011). Prevalence of resistant hypertension in the United States, 2003–2008. Hypertension.

[26] Daugherty SL, Powers JD, Magid DJ, Tavel HM, Masoudi FA, Margolis KL (2012). Incidence and prognosis of resistant hypertension in hypertensive patients. Circulation.

[27] Charan J, Chaudhari M, Mulla S, Reljic T, Mhaskar R, Kumar A (2017). Pharmacotherapy for resistant hypertension in adults.

[28] Eirin A, Textor SC, Lerman LO (2016). Emerging concepts for patients with treatment-resistant hypertension. Trends Cardiovasc Med.

[29] Vemulapalli S, Deng L, Patel MR, Kilgore ML, Jones WS, Curtis LH (2017). National patterns in intensity and frequency of outpatient care for apparent treatment-resistant hypertension. Am Heart J.

[30] Sternlicht H, Bakris GL (2017). Resistant hypertension: a refractory disease or refractory patient. Hypertension.

[31] Chalmers I, Bracken MB, Djulbegovic B, Garattini S, Grant J, Gülmezoglu AM (2014). How to increase value and reduce waste when research priorities are set. Lancet.

[32] Taler SJ, Textor SC, Augustine JE (2002). Resistant hypertension: comparing hemodynamic management to specialist care. Hypertension.

[33] Gaddam KK, Nishizaka MK, Pratt-Ubunama MN, Pimenta E, Aban I, Oparil S (2008). Characterization of resistant hypertension: association between resistant hypertension, aldosterone, and persistent intravascular volume expansion. Arch Intern Med.

[34] Pimenta E, Gaddam KK, Oparil S, Aban I, Husain S, Dell’Italia LJ (2009). Effects of dietary sodium reduction on blood pressure in subjects with resistant hypertension: results from a randomized trial. Hypertension.

[35] Eide IK, Torjesen PA, Drolsum A, Babovic A, Lilledahl NP (2004). Low-renin status in therapy-resistant hypertension: a clue to efficient treatment. J Hypertens.

[36] Shimosawa T (2013). Salt, the renin-angiotensin-aldosterone system and resistant hypertension. Hypertens Res.

[37] Agarwal R (2012). Resistant hypertension and the neglected antihypertensive: sodium restriction. Nephrol Dial Transplant.

[38] Tsioufis C, Kordalis A, Flessas D, Anastasopoulos I, Tsiachris D, Papademetriou V (2011). Pathophysiology of resistant hypertension: the role of sympathetic nervous system. Int J Hypertens.

[39] Dudenbostel T, Acelajado MC, Pisoni R, Li P, Oparil S, Calhoun DA (2015). Refractory hypertension: evidence of heightened sympathetic activity as a cause of antihypertensive treatment failure. Hypertension.

[40] Ouzan J, Perault C, Lincoff AM, Carre E, Mertes M (2002). The role of spironolactone in the treatment of patients with refractory hypertension. Am J Hypertens.

[41] Mahmud A, Feely J (2005). Aldosterone-to-renin ratio, arterial stiffness, and the response to aldosterone antagonism in essential hypertension. Am J Hypertens.

[42] Mahmud A, Mahgoub M, Hall M, Feely J (2005). Does aldosterone-to-renin ratio predict the antihypertensive effect of the aldosterone antagonist spironolactone?. Am J Hypertens.

[43] Gaddam KK, Pimenta E, Husain S, Calhoun DA (2009). Aldosterone and cardiovascular disease. Curr Probl Cardiol.

[44] Pimenta E, Calhoun DA (2007). Resistant hypertension and aldosteronism. Curr Hypertens Rep.

[45] Wang C, Xiong B, Huang J (2016). Efficacy and safety of spironolactone in patients with resistant hypertension: a meta-analysis of randomised controlled trials. Heart Lung Circ.

[46] Bobrie G, Frank M, Azizi M, Peyrard S, Boutouyrie P, Chatellier G (2012). Sequential nephron blockade versus sequential renin-angiotensin system blockade in resistant hypertension: a prospective, randomized, open blinded endpoint study. J Hypertens.

[47] Beaussier H, Boutouyrie P, Bobrie G, Frank M, Laurent S, Coudore F (2015). True antihypertensive efficacy of sequential nephron blockade in patients with resistant hypertension and confirmed medication adherence. J Hypertens.

[48] Dudenbostel T, Calhoun DA (2017). Use of aldosterone antagonists for treatment of uncontrolled resistant hypertension. Am J Hypertens.

[49] Pimenta E, Gaddam KK, Oparil S (2008). Mechanisms and treatment of resistant hypertension. J Clin Hypertens (Greenwich).

[50] Hur E, Ozisik M, Ural C, Yildiz G, Magden K, Kose SB (2014). Hypervolemia for hypertension pathophysiology: a population-based study. Biomed Res Int.

[51] Laragh JH, Sealey JE (2011). The plasma renin test reveals the contribution of body sodium-volume content (V) and renin-angiotensin (R) vasoconstriction to long-term blood pressure. Am J Hypertens.

[52] Li P, Nader M, Arunagiri K, Papademetriou V (2016). Device-based therapy for drug-resistant hypertension: an update. Curr Hypertens Rep.

[53] Ng FL, Saxena M, Mahfoud F, Pathak A, Lobo MD (2016). Device-based therapy for hypertension. Curr Hypertens Rep.

[54] Blumenfeld JD, Sealey JE, Mann SJ, Bragat A, Marion R, Pecker MS (1999). Beta-adrenergic receptor blockade as a therapeutic approach for suppressing the renin-angiotensin-aldosterone system in normotensive and hypertensive subjects. Am J Hypertens.

[55] Azizi M, Menard J (2004). Combined blockade of the renin-angiotensin system with angiotensin-converting enzyme inhibitors and angiotensin II type 1 receptor antagonists. Circulation.

[56] Nobre F. et al. VI Brazilian guidelines on hypertension. Arq Bras Cardiol. 2010;95(1 Suppl):1–51. http://dx.doi.org/10.1590/S0066-782X2010001700001. 21085756

[57] Nobre F, Mion Jr. D, Gomes MAM, et al. V Guidelines for ambulatory blood pressure monitoring (ABPM) and III Guidelines for home blood pressure monitoring (HBPM). Arq Bras Cardiol. 2011;97(3 Suppl)3:1–24. http://dx.doi.org/10.1590/S0066-782X2011001800001. 22262107

