# Supplementary figures and images for: Resistant Hypertension On Treatment (ResHypOT): sequential nephron blockade compared to dual blockade of the renin-angiotensin-aldosterone system plus bisoprolol in the treatment of resistant arterial hypertension – study protocol for a randomized controlled trial
Source: Trials. 2018 Feb 12;19:101. doi: 10.1186/s13063-017-2343-3 (PMC5810004; doi:10.1186/s13063-017-2343-3)

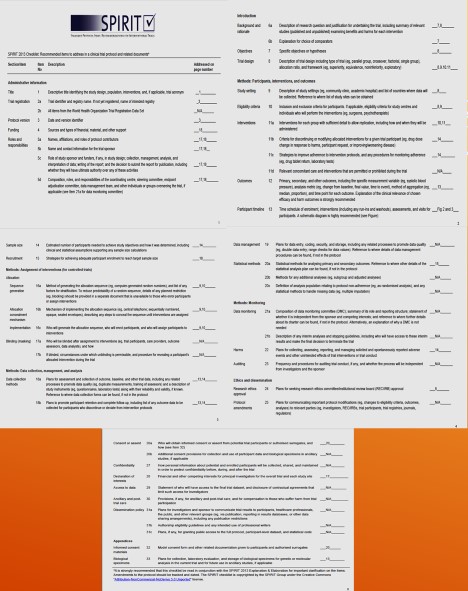

Supplement: Supplementary file 1 — Free and clear term-compliance for research project's participation. (JPG 75 kb) [file 13063_2017_2343_MOESM1_ESM.jpg]
