# Supplementary material for: Resistant Hypertension On Treatment (ResHypOT): sequential nephron blockade compared to dual blockade of the renin-angiotensin-aldosterone system plus bisoprolol in the treatment of resistant arterial hypertension – study protocol for a randomized controlled trial
Source: Trials. 2018 Feb 12;19:101. doi: 10.1186/s13063-017-2343-3 (PMC5810004; doi:10.1186/s13063-017-2343-3)
Supplement: Supplementary file 2 — Research Ethics Committee. Approval Nº 870 093. (PDF 180 kb) [file 13063_2017_2343_MOESM2_ESM.pdf]

ANNEX I

FAMERP
Faculdade de Medicina de São José do Rio Preto

Faculdade de Medicina de São José do Rio Preto

State authority - Law No. 8899 of 09/27/94

(Recognized by Federal Decree No. 74179 of 6/14/74)

Brigadeiro Faria Lima, 5416 -. CEP 15090-000

São José do Rio Preto - São Paulo - Brazil

FREE AND CLEAR TERM-COMPLIANCE FOR RESEARCH PROJECT''S PARTICPATION

Project: **Resistant Hypertension On Treatment (ResHypOT), Sequential nephron blockade compared to dual blockade of the renin-angiotensin-aldosterone system plus bisoprolol in the treatment of resistant arterial hypertension: study protocol for a randomized controlled trial.**

Study objectives:

We are conducting a study to find out the sequence of medicine that is best to treat hypertension resistant or difficult to control: sequential use of diuretics or use of two drugs to block the renin-angiotensin system.

You are being invited to participate. Read the explanations below and request all the information you want to decide whether to participate or not.

How the study:will be conducted

To participate in the study **Sequential nephron blockade compared to dual blockade of the renin-angiotensin-aldosterone system** a period of confirmation of their blood pressure levels is necessary. During this period all participants will be advised to follow healthy lifestyle measures, along with the use of standard therapy.

What are lifestyle measures

Lifestyle measures are behaviors that benefit the overall health and especially prevent cardiovascular diseases such as heart attack, stroke and diabetes. They are recommended for all people and mainly those who have risk factors that increase the chance of having some of them. These measures involve physical exercise, healthy eating, weight control, not smoking and not drinking.

Objectives of the lifestyle measure phases

All participants in the Study **Sequential nephron blockade compared to dual blockade of the renin-angiotensin-aldosterone system** before the intervention phase, should follow the lifestyle measures for three months, and at the end will have blood pressure measured again to confirm whether the blood pressure levels are normalized or not. Accord to your pressure, You will participate in one arm of the study or if it is normal, it will not be eligible to participate in the next phase.

How it will be performed

You will receive guidance on physical activity practice, including activity type and length exercise; on diet to maintain or lose weight, guidelines that can help you quit smoking if your case and reducing the use of alcohol.

When you seeing a doctor 3 months, you will be carried out evaluation of blood pressure with automatic device. Questions will be asked on living habits and measuring weight, height, waist, hip again.

Drug treatment phase

Participants of resistant hypertension will be randomized to receive a sequence of diuretics or a sequence blockers of the renin-angiotensin system, which is taken once daily in the morning. Diuretics eliminate excess of water and salt from the body by decreasing the pressure. Blockers of the renin-angiotensin system cause relaxation of the arteries which reduce the pressure.

They will be held six visits at monthly intervals, the first one is to confirm the diagnosis, the other four are revisions and the last one is to check the results achieved. During the visits, it will be held physical examination and blood pressure with automatic device. Also questions will be asked about your health and lifestyle habits average, weight, waist and hips. An electrocardiography should be performed and along with blood and urine tests at the beginning and end of the study. In addition to the study drugs, the participant will receive non-pharmacological guidance to improve blood pressure control.

The blood collected will be stored and in case of further analysis we will be required a new term-compliance specifically for their achievement.

Risks and discomforts

Verification of pressure causes some discomfort when the device fills to measure the pressure and presses the arm. There may be a small bruise at the site of blood collection.

The drugs used in this study are long for the treatment of hypertension and are considered safe. The study risks are due to the possibility of occurrence of side effects of medications. The most common side effects are muscle cramps, dry mouth, increased urine volume and less frequently increased glucose and uric acid and usually do not require discontinuation of treatment.

There may also be photosensitivity and therefore should be avoided excessive sun exposure. In the event of side effects or problems related to their participation in the study, the researchers and the institution will give you free of charge medical assistance. Pregnant women cannot participate and if pregnancy occurs, should be immediately reported to researchers and the participant will be removed from the study and will remain in follow-up until delivery.

Benefits to participate

There will be no remuneration for participation in the study, but the transportation costs will be funded by research. As a benefit, the participant will receive clinical evaluation and guidance on lifestyle that contribute to a healthy lifestyle. Participants will be collaborating so that the risk of cardiovascular disease is reduced in the population.

Privacy and confidentiality

It is ensured the anonymity of the participants. The results will be published in scientific papers without study participants are identified.

By agreeing to participate in the study, the participant will be committing to follow the study guidelines and attend the planned visits. If you change your mind during the study you can give up participating at any time, but they must attend to conduct the final visit of the study. Refusal to also participate in will not affect

anything in future care in the institution. If you have any questions, you should ask before deciding.

This project was approved by the Ethics in Research of the Faculty of Medicine of São José do Rio Preto and can be contacted on (17) 3201-5813. The researcher is responsible for the study is Prof. Dr. Juan Carlos YugarToledo who can be contacted by phone (17) 3201 -5727 / (17) 9155-5084 or Dr. coordinator. Elizabeth do Espírito Santo Cestário who can be reached by phone (17) 997 186 562

Do you agree to participate?

I declare that I have been given the information described above and agree to participate.

____

Participant

____

Witness

____

Researcher

Place and Date: _____________________ / ________ / 201_
